# Distinct Mechanisms of Target Search by Endonuclease VIII-like DNA Glycosylases

**DOI:** 10.3390/cells11203192

**Published:** 2022-10-11

**Authors:** Evgeniia A. Diatlova, Grigory V. Mechetin, Dmitry O. Zharkov

**Affiliations:** 1 Siberian Branch of the Russian Academy of Sciences Institute of Chemical Biology and Fundamental Medicine, 8 Lavrentieva Ave., 630090 Novosibirsk, Russia; 2 Department of Natural Sciences, Novosibirsk State University, 2 Pirogova St., 630090 Novosibirsk, Russia

**Keywords:** DNA repair, DNA glycosylases, endonuclease VIII, NEIL1, NEIL2, facilitated diffusion, target search

## Abstract

Proteins that recognize specific DNA sequences or structural elements often find their cognate DNA lesions in a processive mode, in which an enzyme binds DNA non-specifically and then slides along the DNA contour by one-dimensional diffusion. Opposite to the processive mechanism is distributive search, when an enzyme binds, samples and releases DNA without significant lateral movement. Many DNA glycosylases, the repair enzymes that excise damaged bases from DNA, use processive search to find their cognate lesions. Here, using a method based on correlated cleavage of multiply damaged oligonucleotide substrates we investigate the mechanism of lesion search by three structurally related DNA glycosylases—bacterial endonuclease VIII (Nei) and its mammalian homologs NEIL1 and NEIL2. Similarly to another homologous enzyme, bacterial formamidopyrimidine–DNA glycosylase, NEIL1 seems to use a processive mode to locate its targets. However, the processivity of Nei was notably lower, and NEIL2 exhibited almost fully distributive action on all types of substrates. Although one-dimensional diffusion is often regarded as a universal search mechanism, our results indicate that even proteins sharing a common fold may be quite different in the ways they locate their targets in DNA.

## 1. Introduction

Many enzymes that recognize specific binding sites in DNA face the problem of finding them quickly and efficiently among a huge excess of competing non-specific DNA. This problem is relevant, in particular, for DNA repair enzymes, transcription factors, and restriction endonucleases [1]. To explain the mechanisms that allow proteins to scan DNA without energy costs, Berg and von Hippel in 1981 proposed four models: (i) sliding, in which the search is carried out by protein diffusion along the DNA chain in a random direction while the DNA–protein complex does not dissociate; (ii) hopping, when the protein moves between closely spaced sites of the DNA chain, always staying at a short distance from it, while the electrostatic interactions between the protein and the DNA are not completely lost; (iii) a distributive mechanism, involving multiple acts of association and dissociation of the DNA–protein complex; and (iv) intersegmental transfer of a protein molecule between sites distant along the DNA contour but accidentally close in space [2]. Sliding and hopping together constitute “facilitated diffusion”, or one-dimensional diffusion, often referred to as processive or correlated search mechanisms. The highest search rate is attained when proteins combine the processive and distributive search. The predominance of a particular mechanism depends on many factors that influence DNA–protein interactions, including the presence of mono- and divalent cations in the solvent shell, competition with other proteins for binding to DNA, etc. [3,4].

DNA repair enzymes target DNA lesions, which constantly appear in DNA due to the action of environmental (UV, ionizing radiation and chemical mutagens) and intracellular (water, reactive oxygen and nitrogen species) damaging factors. This leads to modifications of nucleobases, formation of apurinic/apyrimidinic (AP) sites and single-strand breaks in DNA. Such lesions are usually repaired by the base excision repair system (BER). In the general BER pathway, common for both prokaryotes and eukaryotes, DNA glycosylases initiate the process by searching DNA, recognizing the damaged base and removing it through hydrolysis of the *N*-glycosidic bond between the base and the deoxyribose moiety [5]. AP endonucleases nick DNA at AP sites, either generated by DNA glycosylases or spontaneously formed. DNA polymerases then incorporate an appropriate undamaged nucleotide, or several nucleotides, the 5′-end is processed by a 2′-deoxyribo-5′-phosphate lyase or a flap endonuclease and the nick is sealed by a DNA ligase [5].

The main approaches for studying protein translocation can be divided into single-molecule (fluorescence microscopy or atomic force microscopy) and ensemble (kinetic assays). Several DNA glycosylases have been characterized with respect to their search mechanisms, including bacterial and human uracil–DNA glycosylases (Ung) [6,7,8,9,10,11,12] and mismatched adenine–DNA glycosylases MutY/MUTYH [13,14,15], human thymine-DNA glycosylase [16], alkylpurine–DNA glycosylase [17,18,19], 8-oxoguanine-DNA glycosylase [11,12,20,21,22], bacterial endonucleases III and VIII [23,24] and formamidopyrimidine–DNA glycosylase (Fpg) [13,14,20,21,23,24]. All of them employ, to a various degree, processive search, but different approaches sometimes give quantitatively discordant results, preventing a conclusive description of the search mechanisms.

Fpg and endonuclease VIII (Nei) are two homologous bacterial DNA glycosylases that remove oxidative lesions from DNA. Fpg is specific for damaged purine bases (8-oxoguanine and formamidopyrimidines), whereas Nei excises many oxidatively damaged pyrimidines [25,26]. In the early 2000s, three repair enzymes homologous to Nei were discovered in higher eukaryotes and named NEIL1 (Nei-Like 1), NEIL2 and NEIL3 [27,28,29,30,31,32]. Together with the plant homolog MMH, they form the helix–two-turn–helix (H2TH) structural superfamily characterized by the presence of the namesake DNA-binding motif. These enzymes repair oxidized bases but have overlapping substrate specificities with some other eukaryotic glycosylases (OGG1, NTHL1) and are not essential for the survival of the organism. Despite a number of studies characterizing their biochemistry, it is still an open question what is their main role in the cell. Thus far, it has been shown that NEIL1 interacts with some replication proteins, is up-regulated in the S phase and, therefore, likely repairs damaged bases in double-stranded (ds) DNA during replication [27,33,34,35,36,37]. NEIL2 prefers bubble structures and single-stranded (ss) DNA, interacts with RNA polymerase II and the transcriptional regulator heterogeneous nuclear ribonucleoprotein-U and has been suggested to repair DNA lesions during transcription [38,39,40,41]. NEIL3 shows preference for ssDNA and certain types of interstrand cross-links and likely has some specialized repair function [42,43,44,45].

Mechanisms of lesion search by Nei and NEILs remain poorly studied: diffusion constants for *Escherichia coli* Nei under different conditions were previously obtained using the single-molecule DNA tightrope assay [23,24], and the processivity of human NEIL1 was recently explored using kinetic analysis on a circular plasmid [46]. Here, using a biochemical processivity assay based on the cleavage of an oligonucleotide with two lesions [7,47], we have investigated the contribution of the facilitated diffusion into the lesion search by *E. coli* Nei and murine NEIL1 and NEIL2. Surprisingly, despite the structural similarity between Nei/NEIL1/NEIL2 and Fpg, the mechanisms supporting the processive search by these enzymes seem to be different.

## 2. Materials and Methods

### 2.1. Oligonucleotides and Substrate Preparation

Oligonucleotides were synthesized on an ASM-800 DNA/RNA Synthesizer (Biosset, Novosibirsk, Russia) from commercially available phosphoramidites (Glen Research, Sterling, VA, USA) and purified by electrophoresis in 8% or 20% polyacrylamide gel (PAGE) containing 8 M urea following by reverse-phase chromatography on NenSorb C18 sorbent (DuPont, Wilmington, DE, USA). The sequences of the oligonucleotides are listed in Table 1. When necessary, oligonucleotides were 5′-labeled using γ[^32^P]-ATP (SB RAS ICBFM Laboratory of Biotechnology, Novosibirsk, Russia) and T4 polynucleotide kinase (Biosan, Novosibirsk, Russia) according to the manufacturer’s protocol.

The general scheme of preparation substrates for the correlated cleavage assay was described previously [7,47]. The 40-mer ds substrates X1-X2//comG with two damaged bases were prepared by ligation of [^32^P]-X2 with X1, both annealed to a 1.5-fold molar excess of the complementary 40-mer comG. The mixture was supplemented with T4 DNA ligase (New England Biolabs, Ipswich, MA, USA) and 2 mM ATP, and incubated overnight at 4 °C. The reaction product was purified by electrophoresis in 20% polyacrylamide gel containing 8 M urea and annealed to a 1.5-fold excess of the comG complementary strand.

The 40-mer ss substrate, X1-X2, was prepared similarly to the 40-mer ds substrate but with a 30-mer complementary strand com30 used for annealing to achieve complete separation of the scaffold strand during gel electrophoresis. To obtain the 40-mer bubble substrate, the purified ss 40-mer was annealed to the comBub complementary strand.

The 41-mer ds substrate with 20 bp between two damaged bases, (X1+1)-X2//comG+1, was prepared as above by annealing 5′-[^32^P]-X2 with X1+1 and a 41-mer oligonucleotide scaffold comG+1. The 61-mer ds substrate with 40 bp between two lesions, X1-L40-X2//com40, was prepared by annealing [^32^P]-X2, X1, and a 21-mer 5′-phosphorylated spacer L40 forming an intervening sequence between X1 and X2, with a 61-mer scaffold com40. The 81-mer ds substrate with 60 bp between two lesions, X1-L60-X2//comG60(1)-comG60(2), was prepared by annealing [^32^P]-X2, X1 and a 41-mer 5′-phosphorylated spacer L60, with two scaffold oligonucleotides: a 5′-phosphorylated comG60(1) and non-phosphorylated comG60(2). The 101-mer ds substrate with 80 bp between two lesions, X1-L80-X2//comG80(1)-comG80(2), was prepared similarly with L80 as a spacer and comG80(1) and comG80(2), forming a ligation scaffold. The ligation was performed as described above, and the resulting 41-, 61-, 81- and 101-mer ds oligonucleotides were purified by electrophoresis in non-denaturing 8% polyacrylamide gel at 4 °C. Schematic representations of all substrates used in this work are shown in Table 2.

### 2.2. Enzymes

Full-length murine NEIL1 and NEIL2 carrying a C-terminal His_6_ tag were overexpressed and purified as described [32,48]. For purification of full-length, non-tagged *E. coli* Nei, the pET24b-Nei plasmid [49] was transformed into *E. coli* BL21(DE3) strain. The cells were grown in 2 L of the LB medium containing 100 µg/mL kanamycin at 37 °C to A_595_ = 0.6 and induced with 1 mM isopropyl β-D-1-thiogalactopyranoside for 3 h. The cells were collected by centrifugation at 4 °C, resuspended in 40 mL of the lysis buffer (10 mM Tris–HCl (pH 8.0), 1 mM ethylenediaminetetraacetic acid (EDTA), 500 mM NaCl and 1 mM phenylmethylsulfonyl fluoride) and lysed by sonication. Cell debris was separated by centrifugation at 15,000× *g* for 20 min at 4 °C; then, the supernatant was treated with ammonium sulfate (80% saturation) for 2 h at 4 °C, and centrifuged again. The protein pellet was dissolved in 200 mL of Buffer A (25 mM HEPES–NaOH (pH 7.5), 1 mM EDTA and 1 mM dithiothreitol (DTT)) and the solution was passed through a 0.45 µm filter (Merck Millipore, Burlington, MA, USA), loaded onto a 25-mL SP Sepharose column (GE Healthcare, Chicago, IL, USA) and equilibrated in the same buffer. The bound protein was eluted with a gradient of 0–1000 mM NaCl in Buffer A. Fractions containing the target protein were identified by SDS-PAGE with Coomassie Blue staining. The fractions were diluted with Buffer A to ~50 mM NaCl, loaded onto a 5 mL HiTrap heparin Sepharose column (GE Healthcare), and the bound protein was eluted with a 25 mL gradient of 0–1000 mM NaCl in Buffer A. The fractions containing the target protein were dialyzed overnight against the buffer, containing 20 mM sodium phosphate (pH 7.5), 400 mM NaCl, 1 mM EDTA, 1 mM DTT and 50% glycerol, and stored at −20 °C.

### 2.3. Steady-State Kinetics

Kinetic parameters of Nei, NEIL1 and NEIL2 cleavage of 20-mer substrates X1/C1 and X2/C2 were obtained using AP sites as the lesions. To create AP sites, uracil-carrying substrates (100 pmol) were treated with 10 U of *E. coli* Ung (SibEnzyme, Novosibirsk, Russia) for 10 min at 37 °C immediately before use. The reaction mixture (10 μL) contained 20 mM Tris–HCl (pH 7.5), 1 mM EDTA, 1 mM DTT, 5–500 nM substrate and Nei (5 nM), NEIL1 (5 nM) or NEIL2 (10 nM). After 5 min at 37 °C, 5 μL of formamide-containing dye (80% formamide, 20 mM EDTA, 0.1% xylene cyanol and 0.1% bromophenol blue) was added, and the mixtures were heated for 2 min at 95 °C. The reaction products were resolved by 20% denaturing PAGE and visualized by phosphorimaging (Typhoon FLA 9500, GE Healthcare). The images were quantified using Quantity One v4.6.8 software (Bio-Rad Laboratories, Hercules, CA, USA). *K*_M_ and *V*_max_ were calculated by non-linear fitting to the Michaelis–Menten equation using SigmaPlot v11.0 (Systat Software, Frankfurt am Main, Germany).

### 2.4. Correlated Cleavage Assay

The correlated cleavage assay protocols were similar to the ones described in [7,47]. The concentrations of the substrates were taken in an excess, and the concentrations of the enzymes were optimized to operate in the nearly-linear part of product accumulation time courses. The reaction mixtures (25 μL) contained the appropriate 50 nM substrate, 20 mM Tris–HCl (pH 7.5), 1 mM EDTA, 1 mM DTT, 0–200 mM KCl and 0–20 mM MgCl_2_. Reactions with NEIL2 on ss and bubble substrates did not contain salt. Dependence of cleavage by Nei and NEIL1 on the substrate length was studied at 25 mM KCl. The reactions were initiated by adding Nei (5 nM), NEIL1 (5 nM) or NEIL2 (10 nM) at 37 °C. At time points 0.5–10 min, aliquots (2.5 μL) were withdrawn, quenched with an equal volume of FDLB and heated for 2 min at 95 °C. The reaction products were resolved by 20% or 8% denaturing PAGE and visualized by phosphorimaging as above. The probability of correlated cleavage (*P*_cc_) was calculated as *P*_cc_ = *v*_P3_/(*v*_P1_ + *v*_P2_ + *v*_P3_), where *v*_P1_ and *v*_P2_ are initial rates of accumulation of the products of cleavage at one of the sites and *v*_P3_ is the initial rate of accumulation of the double cleavage product.

### 2.5. Computational Analysis

Structure-assisted alignment of protein sequences was performed using Promals3D [50]. To calculate the electrostatic potential, DNA chains were removed from the structures of human NEIL1 (Protein Data Bank ID 5ITU [51]) and *E. coli* Nei (1K3W [52]), and the resulting protein structures were processed using PDB2PQR v3.5.2 and Adaptive Poisson-Boltzmann Solver (APBS v3.4.1) [53] at pH 7.5 and 0.01 M mobile charged ions (radius 2.0 Å). PyMol (Schrödinger, New York, NY, USA) was used for structural visualization and figure preparation.

## 3. Results

### 3.1. Steady-State Kinetics of Nei, NEIL1 and NEIL2 on Individual Target Sites

**Figure 1:**
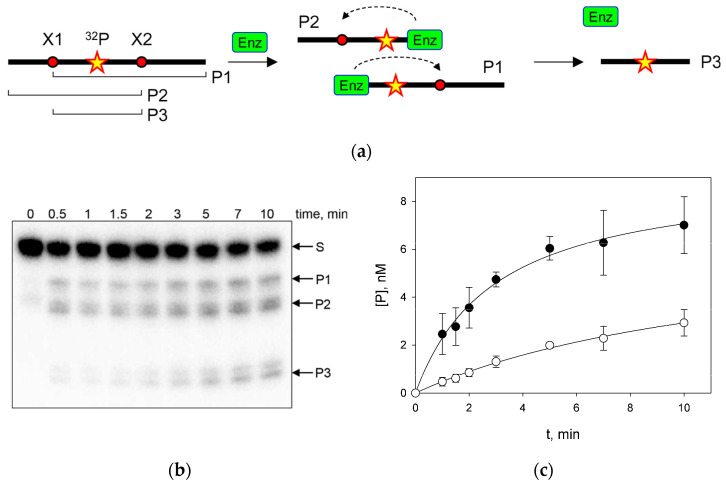
(**a**) General scheme of the correlated cleavage assay. Red dots indicate the damaged sites, star indicates a ^32^P phosphate. (**b**) A representative gel showing the time course of a 40-mer substrate cleavage by Nei at 0 mM KCl. No enzyme was added at the 0 min time point. Arrows indicate positions of the 40-mer substrate and the products of different lengths (P1, 32-mer; P2, 27-mer; P3, 19-mer). (**c**) Time course of accumulation of P1 + P2 (black circles) and P3 (white circles) during the cleavage of a 40-mer substrate by Nei at 0 mM KCl. Mean ± s.d. of four independent experiments are shown.

To study the processive search by Nei and NEILs, we have used a method based on measuring the probability of correlated cleavage of the oligonucleotide substrate containing two lesions [6,7,47]. When the substrate is in excess, full dissociation of the enzyme–product complex after the cleavage at one site makes re-association with the same substrate molecule a rare event, and double cleavage in the initial phase of the reaction reflects the processive transfer of the enzyme between the target sites (Figure 1a). A 40-mer substrate was obtained by ligating two 20-mers with the lesion embedded into identical DNA stretches in order to minimize sequence-dependent effects. To ensure that enzymes would recognize both targets equally well, Michaelis–Menten kinetics were performed on ds 20-mers (X1/C1 and X2/C2). Table 3 shows the kinetic parameters for 20-mers containing an AP site as a lesion universally recognized by Nei, NEIL1 and NEIL2. *K*_M_ and *V*_max_ of Nei and NEIL2 coincide for both substrates within the error margin. For NEIL1, enzyme saturation by the substrate could not be achieved, and the *V*_max_/*K*_M_ ratio was determined instead of the individual *K*_M_ and *V*_max_ values; the *V*_max_/*K*_M_ ratio was similar for both 20-mers. Thus, the interaction of Nei, NEIL1 and NEIL2 with both target sites is similar.

### 3.2. Correlated Cleavage of Substrates

To assess the processivity of Nei, NEIL1 and NEIL2, we used a substrate containing two 5-hydroxyuracil (OHU) residues. OHU is removed by these enzymes with different efficiencies [28,29,31,54] and is much more stable than the AP site thus facilitating preparation of the substrate and avoiding competition with Ung, which is used for AP site preparation in situ. The substrate is designed to produce bands of different mobility upon a single-hit cleavage at either damaged site. Two cleavage products at one of the sites (P1 and P2) and a cleavage product at both sites (P3) were easily observed (Figure 1b,c). From the ratio of the initial P3 accumulation rate to the total accumulation rate of all products, we calculated the probability of correlated cleavage (*P*_cc_), which, if the substrate is in excess, depends only on the nature of the studied enzyme, buffer composition and distance between lesions.

There are several factors that can affect the efficiency of facilitated diffusion, in particular, the presence of mono- and divalent metal ions in the medium [2,3,4]. DNA is always surrounded by a shell of counterions, with monovalent cations usually associated with the major DNA groove and interacting electrostatically, and Mg^2+^ cations forming coordination bonds in both grooves with backbone phosphates and with G in the major groove [55,56]. During diffusion of a protein along DNA, counterions must be displaced by the protein, which characteristically leads to a decrease in the diffusion constant with increasing ionic strength [2,3,4]. The dependence of the efficiency of protein translocation on the cation concentration is often used to prove that a protein searches for its target by one-dimensional scanning rather than in a distributive manner [3,57]. In particular, reduced one-dimensional search with increasing salt concentration was demonstrated for Fpg in both single-molecule and ensemble assays [13,20,21,23].

**Figure 2:**
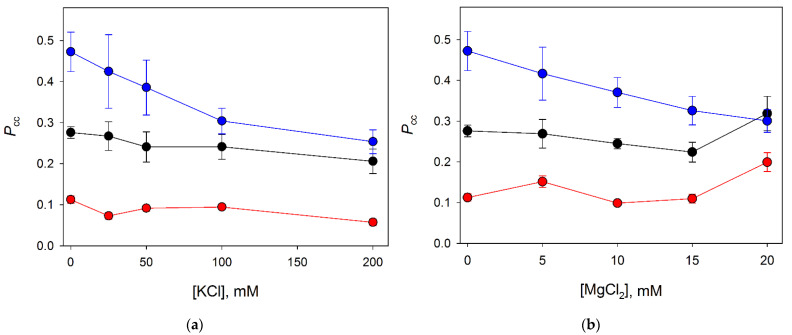
Effect of KCl (**a**) and MgCl_2_ (**b**) on *P*_cc_ of Nei (black circles), NEIL1 (blue circles) and NEIL2 (red circles). Mean ± s.d. of four independent experiments are shown.

We have studied the effect of K^+^ and Mg^2+^ cations on *P*_cc_ of Nei, NEIL1 and NEIL2 by varying the concentrations of KCl from 0 to 200 mM (Figure 2a) and MgCl_2_ from 0 to 25 mM (Figure 2b). Quite unexpectedly, of all three enzymes, only NEIL1 was consistent with a processive search: its *P*_cc_ value reached 0.48 in the absence of cations other than Tris in the buffer, and decreased with increasing ion concentrations of K^+^ or Mg^2+^ (Figure 2, blue plots). NEIL2 hardly used processive mechanisms, its *P*_cc_ values being about 0.1 over the entire range of cation concentrations (Figure 2, red plots). Nei behaved in an intermediate way, its *P*_cc_ values demonstrating a very shallow (but statistically significant) descent from 0.28 to 0.21 upon increasing KCl and largely insensitive to MgCl_2_ (Figure 2, black plots). It is likely that such a profile indicates some features of the interaction of Nei and NEIL2 with DNA, indicating perhaps that their binding is mostly stabilized by non-electrostatic interactions, such as hydrophobic or van der Waals interactions. At 20 mM MgCl_2_, *P*_cc_ increased noticeably for both Nei and NEIL2, which might be due to better stabilization of the duplex or the protein by Mg^2+^ ions. In general, the processivity of Nei, NEIL1 and NEIL2 was noticeably lower than that of the structurally related *E. coli* Fpg, for which, in exactly the same substrate system and reaction conditions, *P*_cc_ values up to 0.9 have been reported [21].

### 3.3. Distance Dependence of the Correlated Cleavage

**Figure 3:**
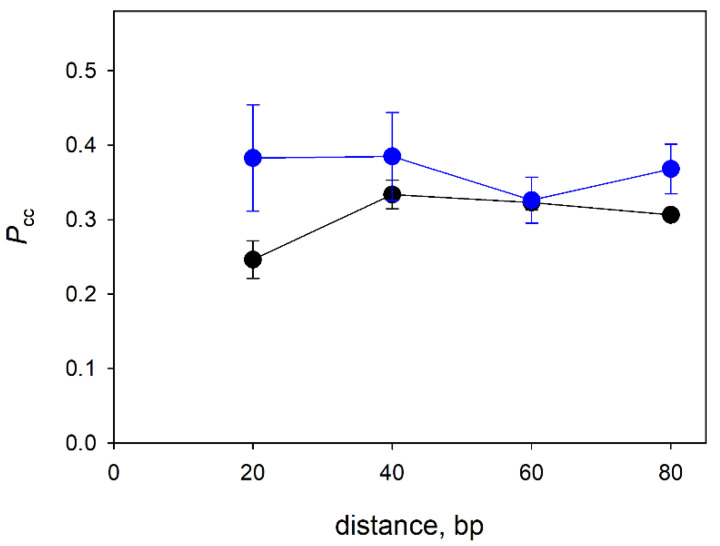
Dependence of *P*_cc_ of Nei (black circles) and NEIL1 (blue circles) on the distance between the damaged sites in the presence of 25 mM KCl. Mean ± s.d. of three independent experiments are shown.

Processive target search in DNA is essentially a random walk with a finite probability of loss of the protein at each step [58]. Thus, the probability of correlated cleavage should decrease with longer distances between the lesion sites, as was shown for *E. coli* Ung [6,8,47]. To estimate how far Nei and NEIL1 can move in a processive manner along DNA before dissociation, we have determined *P*_cc_ on substrates where two OHU residues were separated by 20, 40, 60 or 80 bp. NEIL2 was not included because of its low processivity even at a short distance (see above). Much to our surprise, there was no noticeable decrease in the *P*_cc_ values over the entire range of intersite distances (Figure 3). In a recent report on the lesion search in plasmid substrates by human NEIL1, the estimated mean translocation distance was approximately 80 bp [46]. Given that NEIL1 here and in [46] showed a typical salt dependence profile, it is possible that NEIL1 is indeed processive and a decrease in *P*_cc_ would be observed at distances > 80 bp, which are not easily achieved in an oligonucleotide-based system. On the other hand, *P*_cc_ of Nei apparently did not depend on the intersite distance, which, together with weak salt dependence, is indicative of the low processivity of target search by this enzyme (see the Discussion for factors affecting the bulk processivity observed in double-cleavage experiments).

### 3.4. Processivity of NEIL2 on Substrates of Different Structure

**Figure 4:**
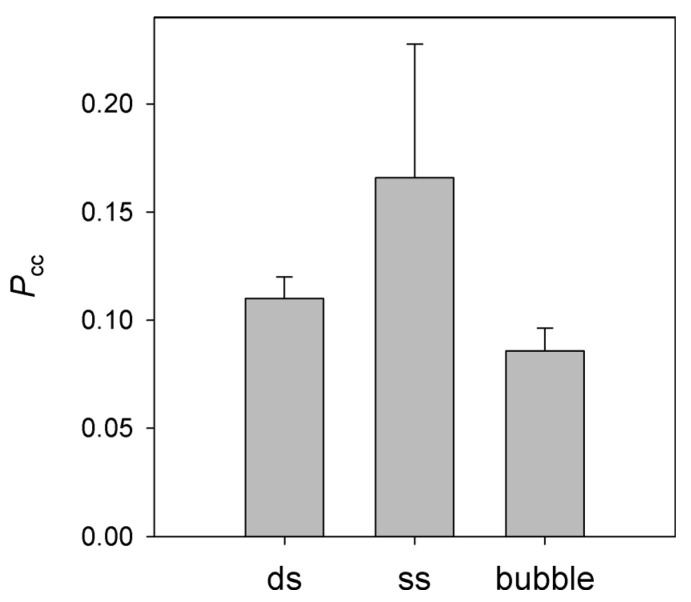
Correlated cleavage of double-stranded (ds), single-stranded (ss) and bubble DNA substrates by NEIL2. Mean ± s.d. of three independent experiments are shown.

Since dsDNA is not an optimal substrate for NEIL2, which prefers bubble and single-stranded substrates [38,41], we have inquired whether the correlated cleavage by NEIL2 may be higher in these alternative substrates. In the bubble substrate, the OHU residues were located at both ds/ss junctions of a 19-mer bubble; such substrates are efficiently processed by NEIL2 [41]. As can be seen in Figure 4, there was a tendency towards higher *P*_cc_ in the single-stranded substrate, which, however, did not reach statistical significance. No significant difference in *P*_cc_ was observed for the ds and bubble substrates. Together with the independence of *P*_cc_ on K^+^ and Mg^2+^ concentrations and the overall low *P*_cc_ values, these findings strongly suggest that NEIL2 uses a distributive mechanism to search for its target sites in DNA.

## 4. Discussion

Many proteins that recognize specific sequences, chemical modifications or structural elements in DNA, despite possessing quite different structural folds, share a common mechanism of target search, namely facilitated one-dimensional diffusion along the DNA contour. In particular, such a mechanism was demonstrated for a number of DNA glycosylases, the enzymes that recognize damaged nucleobases and initiate base excision DNA repair [6,7,8,9,10,11,12,13,14,15,16,17,18,19,20,21,22,23,24,46]. Here, we show that in a group of structurally related DNA glycosylases, all belonging to the same H2TH structural superfamily, may vary greatly in their ability to carry out processive lesion search, which, therefore, should not be taken for granted as a universal target location mechanism.

Together with this study, there are now four H2TH DNA glycosylases characterized to any extent with respect to the search mechanism: Fpg, Nei, NEIL1 and NEIL2 [13,14,20,21,23,24,46]. Fpg seems to be the most clear-cut case. In both ensemble [13,21] and single-molecule assays [14,20,23,24], Fpg from *E. coli* and *Geobacillus stearothermophilus* demonstrated considerable processivity. Single-molecule studies report the binding lifetime of Fpg at low salt in the 0.1 s–3.0 s range and the diffusion constant in the 3.5 × 10^5^ bp^2^/s–1.3 × 10^6^ bp^2^/s range. This translates to an average displacement of 265 bp–2790 bp in a single binding event, consistent with very high *P*_cc_ (~0.9) observed in double-cleavage experiments [21]. At the other extreme is NEIL2, which, as we show here, is apparently almost fully distributive with dsDNA, ssDNA and bubble substrates. This is probably not surprising, as NEIL2 is known to be associated with the RNA polymerase II transcription complex [39,40,59], and thus, may not need to search for the lesions on its own, relying instead on a stalled polymerase for target recognition.

Rather unexpectedly, an increase in the distance between two target sites had almost no effect on *P*_cc_ of Nei and NEIL1. This is contrary to what was observed with Ung, which uses processive search surveying ~100 bp per binding event and shows correlated cleavage rapidly declining with distance [6,47]. However, NEIL1 demonstrated a typical salt dependence expected of processive search, with a significant fraction of correlated cleavage even at 200 mM KCl or 20 mM MgCl_2_, whereas the correlated cleavage by Nei only modestly decreased from 0 to 200 mM KCl and was overall lower than the correlated cleavage by NEIL1. We suggest that this behavior may be explained in the framework of a model was proposed by Dunn et al. based on single-molecule “tightrope” experiments on target search in a high-molecular-weight λ phage DNA [23]. The model postulates that protein molecules searching for the target exist as at least two conformational populations: a slowly moving, tightly bound interrogation state, in which the protein probes whether the local properties of DNA correspond to its target, and a non-specific state, in which the protein rapidly slides and easily releases the bound DNA. If the populations are interconverting slowly, *P*_cc_ values in the distance dependence experiment (Figure 3) would reflect the fraction of the enzyme molecules in the interrogation state with characteristic displacement distance considerably exceeding the largest intersite distance (80 bp in our case), while the others are rapidly falling off the DNA molecule. Consequently, ~40% of NEIL1 molecules and ~30% of Nei molecules would be in the interrogation state (Figure 3). Indeed, it is Ung that may be an exception from a common mechanism, since it recognizes uracil bases in spontaneously transiently opened base pairs [60,61], obviating the need for a dedicated interrogation state, whereas other DNA glycosylases, such as Fpg, have to force the target base pair opening [62,63,64,65].

**Figure 5:**
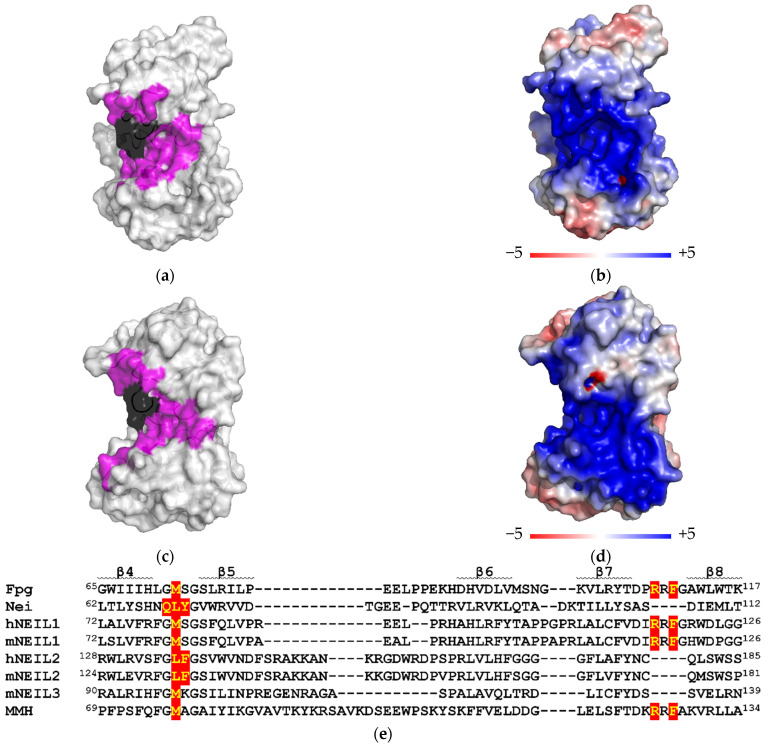
Structures of (**a**,**b**) NEIL1 (5ITU [51]) and (**c**,**d**) Nei (1K3W [52]). In (**a**,**c**), the DNA-binding groove defined as the residues within 4.0 Å from DNA is colored in magenta, and the intercalation triad is in black. In (**b**,**d**), the protein surface is colored according to the charge (blue, positive, red, negative; bar scale is in *k*_B_*T*/*e*_c_ units). (**e**), alignment of a part of the β-sandwich domain of H2TH superfamily proteins (*E. coli* Fpg, *E. coli* Nei, human NEIL1, mouse NEIL1, human NEIL2, mouse NEIL2, mouse NEIL3, *Arabidopsis thaliana* MMH) showing the intercalating triad in red.

Structural data available for Fpg, Nei, NEIL1 and MMH bound to DNA indicate that they all share similar overall organization of protein–DNA interactions [51,52,66,67,68]. The proteins possess an extensive, positively charged DNA-binding groove with a deep pocket, where the damaged base binds after being flipped out of the helix (Figure 5a–d). The H2TH motif and a β-hairpin zinc finger (or, in NEIL1 and MMH, an equivalent β-hairpin lacking a Zn^2+^ ion) bind backbone phosphates near the lesion and sharply kink the DNA axis facilitating the base flip-out. Although no structure of NEIL2 or NEIL3 bound to DNA is available, conservation of these basic elements in the structures of free NEIL2 from gray short-tailed opossum (*Monodelphis domestica*) [69], mouse NEIL3 [43] and the NEIL2/NEIL3-like protein from the giant Mimivirus [70] suggests that they bind DNA in a similar way. However, one prominent difference between Fpg/NEIL1/MMH on the one hand and Nei/NEIL2/NEIL3 on the other hand is the organization of a trio of amino acids that are inserted into the DNA helix to probe for damage and initiate the base eversion (Figure 5e). In Fpg, NEIL1 and MMH, they are absolutely conserved and include a methionine (Met80 in NEIL1) that fills the void left after base eversion, an arginine that recognizes the base opposite to the lesion (Arg117 in NEIL1) and a phenylalanine that wedges between the damaged base pair and the adjacent one to kink DNA (Phe119 in NEIL1). The Met residue comes from a loop between β4 and β5 strands in the N-terminal β-sandwich domain, whereas the Arg and Phe residues come from the β7/β8 loop (Figure 5a,e). In Nei, all three functionally equivalent residues (Leu, Gln and Tyr, respectively) come from the β4/β5 loop, and the β7/β8 loop is missing completely (Figure 5c,e). NEIL2 and NEIL3 retain, respectively, only two residues and one residue of the triad and also lack the β7/β8 loop, which likely reflects their preference for non-duplex DNA substrates. Nei, however, prefers ds substrates to ss and bubble substrates [41]. Nevertheless, the presence of a highly positively charged Arg residue in Fpg and NEIL1 right inside the DNA duplex could be one reason for tighter interaction and more pronounced salt sensitivity in comparison with the uncharged intercalating residues in Nei and NEIL2.

Yet another possibility is that overall lower Nei/NEIL2 processivity could be rooted in differences in the protein structural dynamics. H2TH proteins’ catalytic core consists of two domains, N- and C-terminal, connected with a flexible linker. Regarding the orientation of their domains, Nei and NEIL2 proteins are shown to exist in two conformations, open and closed [52,69,71,72]. It is thought that free Nei and NEIL2 assume an open conformation and close upon DNA binding. Free NEIL1, however, exists in a closed conformation [73]. The structure of free *E. coli* Fpg is unknown; free Fpg from *Neisseria meningitides* was crystallized in an open conformation [74], whereas free Fpg from *Thermus thermophilus* is closed [75]. Thus, Nei and NEIL2 could be intrinsically more prone to opening and DNA release than NEIL1 and Fpg.

In conclusion, regardless of the structural and mechanistic reasons underlying the differences in the processivity of H2TH superfamily DNA glycosylases, our results emphasize that DNA repair proteins, even those sharing a common fold, may be quite variable in the ways they use to search for their targets. Biologically, Fpg, NEIL1 and Nei likely act on their own to find DNA lesions, while NEIL2 most likely relies on an RNA polymerase as a damage sensor, which may explain its lower processivity as an isolated enzyme. Within the constraints imposed by their structure, proteins likely combine processive and distributive modes to achieve the most efficient search in the environment of the eukaryotic nucleus or the bacterial nucleoid. Although there are very few examples of facilitated diffusion studied in living cells [76,77], only the development of suitable in situ assays will give an answer how well the mechanisms deduced from in vitro experiments align with the real kinetics of damage search by DNA repair enzymes.

## Figures and Tables

**Table 1:** Sequences of the oligonucleotides used in this work.

ID	Sequence (5′→3′) ^a,b,c^
X1	TCCCTTC**X**CTCCTTTCCTTC
X2	GGACTTC**X**CTCCTTTCCAGA
C1	GAAGGAAAGGAG**C**GAAGGGA
C2	TCTGGAAAGGAG**C**GAAGTCC
comG	TCTGGAAAGGAG**G**GAAGTCCGAAGGAAAGGAG**G**GAAGGGA
X1+1	TCCCTTC**X**CTCCTTTCCTTCC
comG+1	TCTGGAAAGGAG**G**GAAGTCCGGAAGGAAAGGAG**G**GAAGGGA
L40	pGGACTTTACTTGCGTTAGAGC
comG40	TCTGGAAAGGAG**G**GAAGTCCGCTCTAACGCAAGTAAAGTCCGAAGGAAAGGAG**G**GAAGGGA
L60	pGGACCTTTCATTTGTGCGATCTTTCCTCTCGTTCAGACCTC
comG60(1)	pGATCGCACAAATGAAAGGTCCGAAGGAAAGGAG**G**GAAGGGA
comG60(2)	TCTGGAAAGGAG**G**GAAGTCCGAGGTCTGAACGAGAGGAAA
L80	pGGACCTTTCATTTGTGCGATGAGTGAATTTCGGGATTTAGCTTTCCTCTCGTTCAGACCTC
comG80(1)	pAAATTCACTCATCGCACAAATGAAAGGTCCGAAGGAAAGGAG**G**GAAGGGA
comG80(2)	TCTGGAAAGGAG**G**GAAGTCCGAGGTCTGAACGAGAGGAAAGCTAAATCCCG
comBub	TCTGGAAAGGAGATGGACTAACGAACCCAAGTAGAAGGGA
com30	AAAGGAGCGAAGTCCGAAGGAAAGGAGCGA

^a^ X, uracil (U) or 5-hydroxyuracil (OHU); ^b^ p, 5′-terminal phosphate introduced synthetically; ^c^ Bold underlined letters indicate the target base or the base complementary to it.

**Table 2:** Schemes of oligonucleotide substrates *^a,b^*.

Type of the Experiment			
Steady-state kinetics	Dependence on [K^+^] and [Mg^2+^]	Processivity of NEIL2	Dependence on the distance between the targets
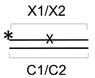	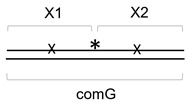	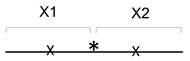	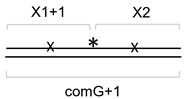
		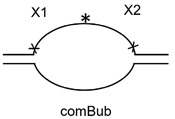	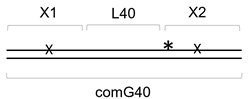
			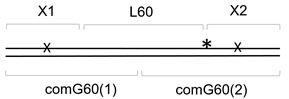
			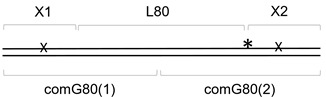

*^a^* See Table 1 for the IDs of the oligonucleotides used to construct the substrates. *^b^* Asterisk indicates the location of the [^32^P] label. X indicates the damaged site.

**Table 3:** Kinetic parameters of cleavage of double-stranded 20-mer oligonucleotides with an AP site by Nei, NEIL1 and NEIL2 ^*a*^.

		*K*_M_, nM	*V*_max_, nM × min^−1^	*V*_max_/*K*_M_, min^−1^
Nei	X1/C1	52 ± 13	10 ± 2	0.20 ± 0.02
	X2/C2	56 ± 17	11 ± 2	0.20 ± 0.02
NEIL1	X1/C1	n/d *^b^*	n/d	0.08 ± 0.01
	X2/C2	n/d	n/d	0.12 ± 0.01
NEIL2	X1/C1	101 ± 22	17 ± 3	0.17 ± 0.01
	X2/C2	138 ± 37	17 ± 3	0.15 ± 0.02

*^a^* Mean ± s.d. of two independent experiments; *^b^* n/d, not determined.

## Data Availability

All data are contained in the paper.
